# Characterization of plant growth promoting rhizobacteria and their benefits on growth and phosphate nutrition of faba bean and wheat

**DOI:** 10.1242/bio.043968

**Published:** 2019-07-05

**Authors:** Noura Bechtaoui, Anas Raklami, Abdel-Ilah Tahiri, Loubna Benidire, Abdelkhalek El Alaoui, Abdelilah Meddich, Michael Göttfert, Khalid Oufdou

**Affiliations:** 1 Laboratory of Biology and Biotechnology of Microorganisms, Faculty of Sciences Semlalia, Cadi Ayyad University, PO Box 2390, Marrakech, Morocco; 2 Laboratory of Biotechnology and Plant Physiology, Faculty of Sciences Semlalia, Cadi Ayyad University, PO Box 2390, Marrakech, Morocco; 3 Technische Universität Dresden, Institut für Genetik, Helmholtzstr. 10, D-01069 Dresden Germany

**Keywords:** PGPR, Rhizobia, *Triticum durum*, *Vicia faba*, Plant improvement, Phosphorus

## Abstract

In recent years, more attention has been paid to plant growth promoting (PGP) rhizobacteria use as a biofertilizer alternative to chemical fertilizers, which might cause damage to the environment. The main objective of this work was to evaluate the field application of PGP bacteria and rhizobial strains on the productivity of two food crops extensively used in Morocco; *Vicia faba* L. and *Triticum durum* L. A field experiment with four treatments was designed: (1) control without inoculation, (2) PGP bacteria alone (P), (3) rhizobia alone (R) and (4) a mixture of PGP-rhizobia (PR). Furthermore, the PGP strains were tested for their ability to solubilize complex mineral phosphorus and potassium and for their production of indole acetic acid and exopolysaccharides. The strains showed several plant growth promoting traits. Field inoculation by these rhizobacteria improved phosphorus uptake and the agronomic parameters of faba bean and wheat plants, such as biomass of shoots and roots, as well as the weight of bean pods and wheat spikes. The most pronounced effect was displayed by rhizobial strains or the combination of PGP-rhizobia. The rhizobacterial inoculation significantly stimulated the growth of both crops and could be used as potential biofertilizers to optimize growth and phosphorus retention capacity.

## Abstract (precis)

**Summary:** We provide phenotypic and molecular characterization of rhizobacterial strains and their effectiveness in the field to boost growth and phosphorus nutrition of wheat and faba bean.

## INTRODUCTION

Phosphorus has a vital role inside the cell as an energetic and structural element, and its deficiency severely affects crop yield. In fact, the problem of phosphorus is not about its abundance because most cultivated land contains a significant amount of phosphorus. Specifically, it is the availability of phosphorus in the soil solution, which is often insufficient and does not cover the nutritional requirement of plants (Walpola and Yoon, 2012). Vance et al. (2003) reported that phosphorus is the most limiting nutrient for crop yield in more than 30% of the world's arable land. As a solution to adjust the phosphate status inside the soil, farmers depend on chemical fertilizers to boost agronomical production. Nevertheless, once a fertilizer is applied to the soil system, the processes of adsorption and precipitation occur, and only about 15–30% of the fertilizer can be absorbed by plant roots (Sharma et al., 2013). On the other hand, López-Arredondo et al. (2014) reported that rock phosphate, apatite and other raw materials used in the manufacture of phosphate fertilizers, which occur in finite deposits mainly in China, the United States and Morocco, are becoming increasingly limited. Therefore, there is an urgent need to search for alternative strategies to enhance crop productivity in poor soils, to secure food production and to improve the efficiency of phosphate fertilizers.

Phosphorus exists in soil solution in micromolar or lower concentrations due to its high reactivity with soil compounds. It is generally complexed with aluminum, iron and other metallic ions in acidic soil or with calcium carbonate in alkaline soil (Gyaneshwar et al., 2002). Making phosphate more available in soil solution is realized by plant growth promoting rhizobacteria (PGPR), which can solubilize both organic and inorganic phosphate forms in soil. Species such as *Bacillus*, *Pseudomonas*, *Acinetobacter*, *Rhizobium* and other bacteria possess the ability to solubilize complex forms of phosphate (Krishnaraj and Dahale, 2014). In addition, they support plant growth via synthesis of phytohormones or other growth-promoting or protecting substances like siderophores and antibiotics. Among PGPR strains, rhizobia are involved in the symbiotic fixation of atmospheric nitrogen with legumes (Satyaprakash et al., 2017). The use of PGPR strains in agricultural practices is strongly encouraged as they may constitute a sustainable solution that can improve the efficiency of chemical fertilizers.

In Morocco and over the world, the available information about the effect of endogenous PGPRs on growth promotion and phosphorus bioavailability under field conditions is generally poor. The expected field outcome is sometimes difficult to achieve due to the complexity of abiotic and biotic factors and their interactions that might influence the bacterial function and subsequently the crop productivity (Bais et al., 2006; Toppo and Tiwari, 2015). It is therefore urgent to find the appropriate combination that can be adapted to the rhizosphere microbiota and improve plant growth under phosphorus limitation. The objective of this study was (1) to isolate and identify endogenous PGPRs from faba bean rhizosphere and (2) to evaluate their effect on plant growth (wheat and faba bean) in field conditions.

## RESULTS

### PGPR characteristics of the rhizobacterial strains

The PGP and rhizobial strains were tested on agar media containing different sources of mineral phosphate (Table 1). The phosphate solubilizing capacity clearly depended on the medium used and also on the variety of complex phosphate. The solubilization of monocalcium and tricalcium phosphate was easier than the solubilization of rock phosphate. After 3 days of inoculation, strains BS17 and PGP27 had already formed visible halos on most of the media and halos were visible on all media after 15 days. For the two rhizobial strains, the onset of halo formation was later and halos were often smaller. The highest DH/DC values for BS17 were observed after 10 days (DH/DC of 4.02 on NH_4_Cl medium) and for PGP27 after 15 days of inoculation on NBRIY medium (DH/DC of 3.03) (Table 1).

**Table 1.**
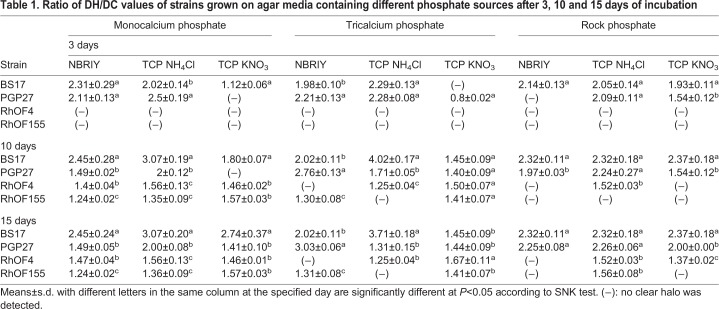
**Ratio of DH/DC values of strains grown on agar media containing different phosphate sources after 3, 10 and 15 days of incubation**

In liquid medium, rhizobial strains produced more available phosphate, even though they had shown lower DH/DC values. The pH in the supernatant turned acidic within the first 24 h (Table 2). The objective to follow the fluctuation of acidity in the broth is that the release of phosphorus is probably due to acids produced by the solubilizing strain (Alikhani et al., 2006).

**Table 2.**
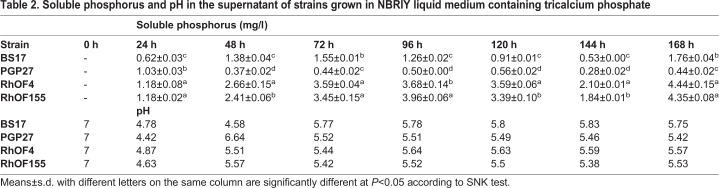
**Soluble phosphorus and pH in the supernatant of strains grown in NBRIY liquid medium containing tricalcium phosphate**

Indole acetic acid is considered as a phytohormone directly promoting plant growth. Production of exopolysaccharides by microorganisms has multiple benefits for plants, e.g. alleviation of drought stress in addition to their contribution to phosphate solubilization (Yi et al., 2008; Grover et al., 2011). Therefore, we determined indole acetic acid and exopolysaccharides produced by the bacterial strains (Table 3). The maximal amount of indole acetic acid was recorded for RhOF155 (290.64 µg/ml) followed by RhOF4 (112.43 µg/ml), whereas PGP27 and BS17 produced lower amounts compared to rhizobial strains. Similarly, the rhizobial strains produced the highest quantities of exopolysaccharides. On the other hand, all strains solubilized potassium with an increase of the DH/DC values during the incubation time. The maximum was registered for PGP27 (3.02) and RhOF4 (2.55) after 144 h of inoculation (Table 3).

**Table 3.**
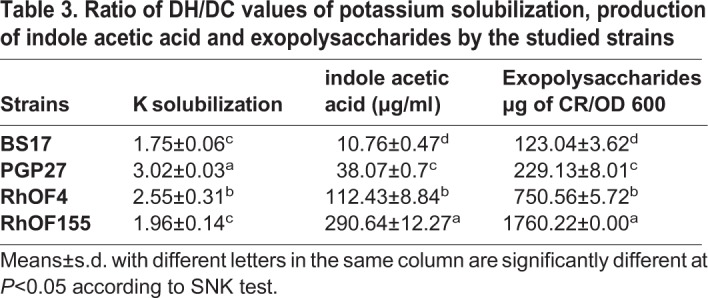
**Ratio of DH/DC values of potassium solubilization, production of indole acetic acid and exopolysaccharides by the studied strains**

**Fig. 1.**
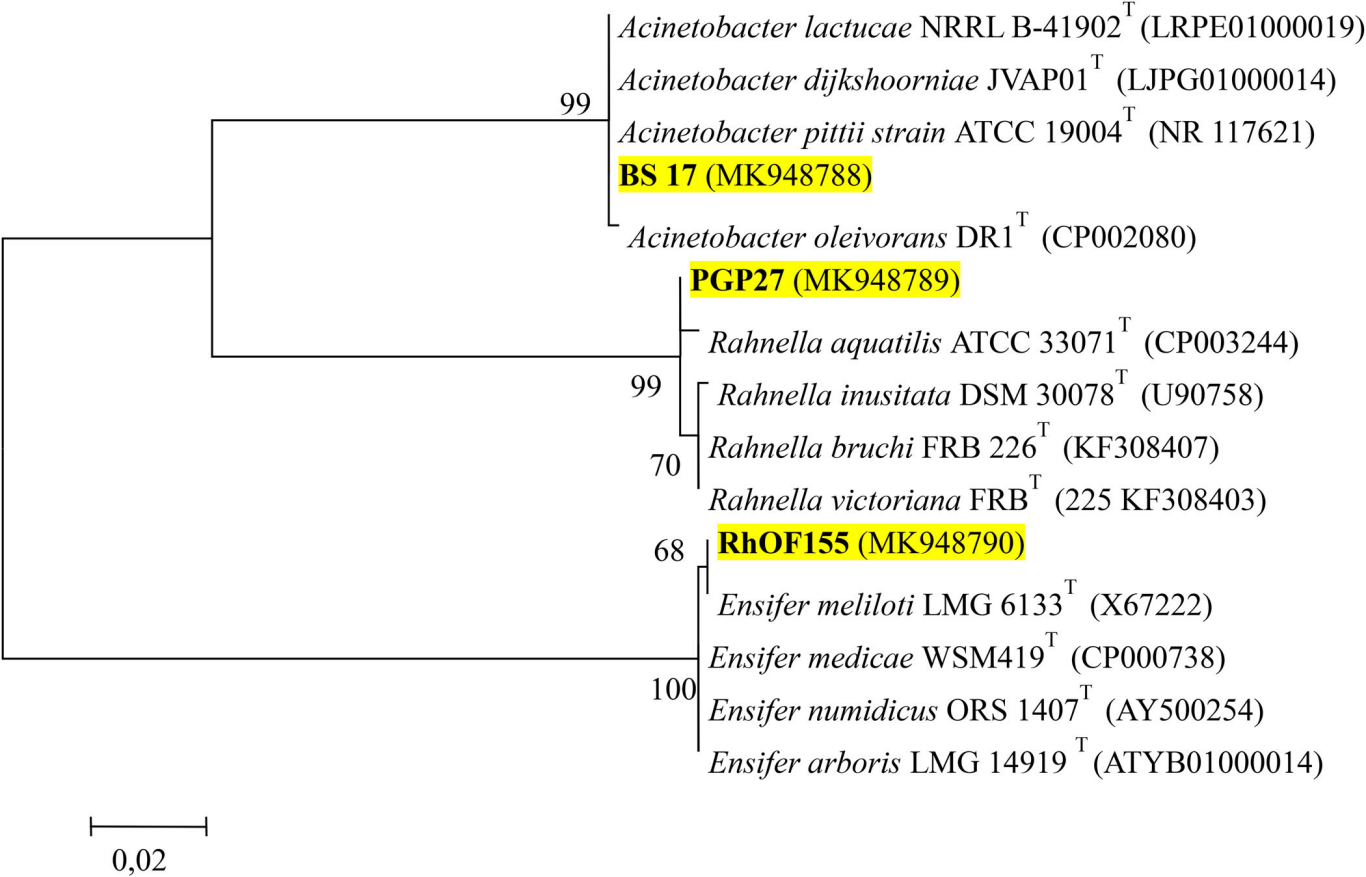
**Maximum Likelihood phylogenetic tree of the strains PGP27, BS17** **and** **RhOF155 based on 16S rDNA gene sequences, showing the position of the strains with regard to related species.** Bootstrap values based on 1500 replications are given at branch points. Numbers in parentheses represent the sequence accession numbers in GenBank. Scale bar: substitutions per nucleotide position.

The molecular characterization of the16S rDNA gene of the strains showed that BS17 is closely related to *Acinetobacter* sp., PGP27 is very similar to the type strain of *Rahnella aquatilis*, whereas RhOF155 is identified as *Ensifer meliloti* (Fig. 1). As for RhOF4, it was already identified by our team as *E. meliloti* (*Sinorhizobium meliloti*) (Benidire et al., 2018).

The 16S rDNA nucleotide sequences determined in this work were submitted to the GenBank database and have been assigned the accession numbers MK948788, MK948789 and MK948790 for BS17, PGP27 and RhOF155, respectively. The sequence of strain RhOF4 was previously deposited in GenBank and is available under accession number MF687953 (Benidire et al., 2018).

### Effects of rhizobacterial inoculation of wheat and faba bean in field conditions

**Fig. 2.**
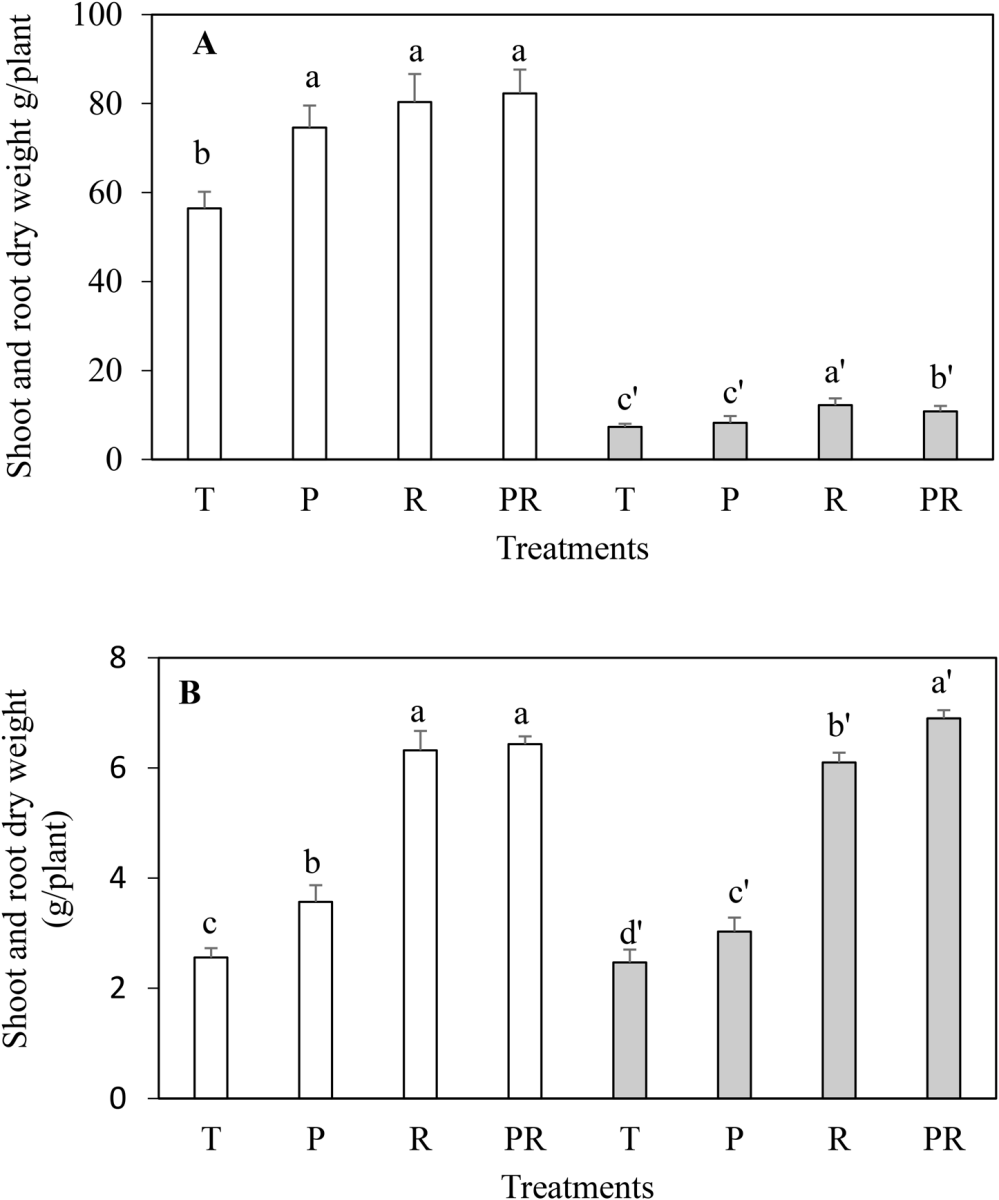
**Shoot dry weight (colored in white) and root dry weight (colored in gray) of faba bean (A) and wheat (B) submitted to different treatments.** T, uninoculated control; P, PGP only (PGP27+BS17); R, rhizobia only (RhOF4+RhOF155); PR, PGP+rhizobia. Means (±s.d.) within the same graphic followed by different letters are significantly different at *P*<0.05.

**Fig. 3.**
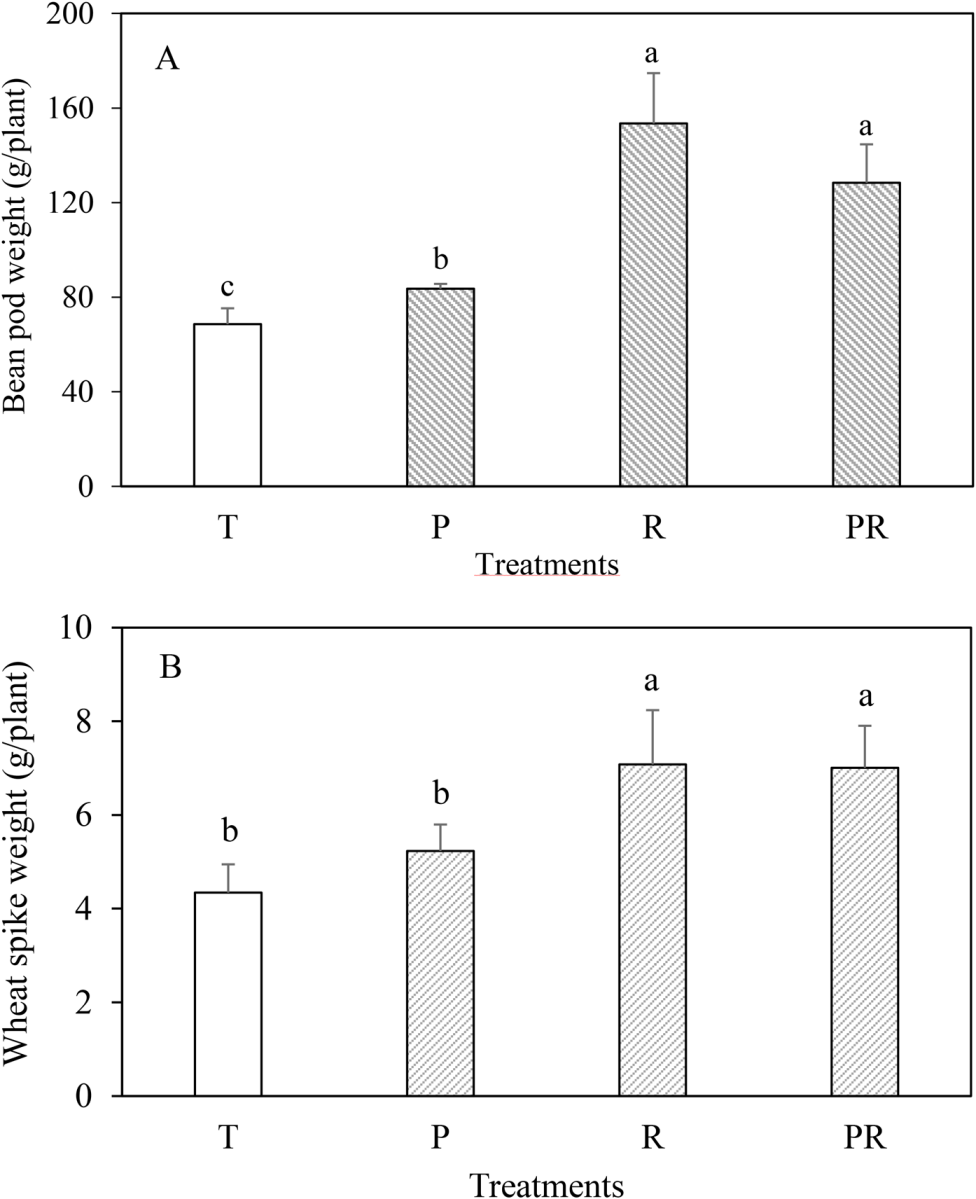
**Green bean pod weight (A) and dry wheat spike weight (B) of plants submitted to different treatments.** T, uninoculated control; P, PGP only (PGP27+BS17); R, rhizobia only (RhOF4+RhOF155); PR, (PGP+rhizobia). Means (±s.d.) within the same graphic followed by different letters are significantly different at *P*<0.05.

To test rhizobacterial plant growth-promoting activity, wheat and faba bean were inoculated with different strain combinations. Inoculation of wheat with rhizobial strains (R) and the mixture PGP-rhizobia (PR), more than doubled shoot and root dry weight compared to the uninoculated control (Fig. 2). Inoculation with the PGP strains (P) also led to a significant increase of shoot dry weight, albeit to a lower extent. For faba bean, shoot dry weight was significantly increased to a comparable level with all bacterial combinations (compared to the uninoculated control), whereas root dry weight increased only with rhizobia or the combination of all strains (Fig. 2). The weight of bean pods and wheat spikes of the inoculated plants were significantly increased confirming the beneficial effect of the selected strains (Fig. 3). Indeed, the inoculation with the rhizobia (R) and the mixture (PR) enhanced faba bean pod weight by 123.78% and 87.21%, respectively, compared to the control. In case of wheat spikes, treatments increased their dry weight by 63.05% and 61.50% in comparison to the uninoculated control. It is likely that an increased phosphorus supply contributed to this plant growth promotion as the phosphorus concentration of the plants also increased if they were inoculated. Highest values were obtained when the plants were inoculated with the mixture PGP-rhizobia (Table 4).

**Table 4.**
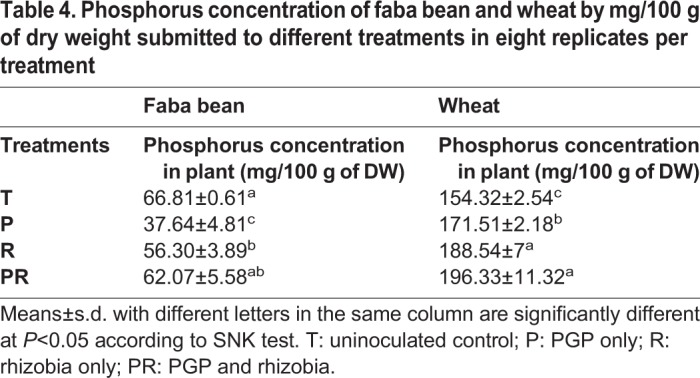
**Phosphorus concentration of faba bean and wheat by mg/100 g of dry weight submitted to different treatments in eight replicates per treatment**

## DISCUSSION

It is known that rhizospheric bacteria provide maintenance and proper distribution of nutrients in the soil (Richardson and Simpson, 2011). Several studies looked into the impact of root exudates on microbial communities. The chemical components of root exudates may deter one organism while attracting others (Chaparro et al., 2012). This makes the direct application of any exogenous microorganism in the field more challenging. The studied PGPR strains are native to the rhizosphere of faba bean and should be competitive in the soil of the Marrakech region while enhancing plant growth.

The *in vitro* analysis of phosphate solubilization for strains PGP27, BS17, RhOF4 and RhOF155 revealed the effect of culture media and the source of complex phosphate on solubilization capacity. Halo formation is generally caused by the release of organic acids, which chelate calcium associated with phosphate and thus make phosphorus more available (Satyaprakash et al., 2017). On the other hand, organic acid production depends strongly on carbon and nitrogen sources. Mardad et al. (2014) found that glucose as carbon source and (NH_4_)_2_SO_4_ as the nitrogen source, significantly increased tricalcium phosphate solubilization. Nitrogen source in the medium may lead to acidification or alkalinization, which in turn influences phosphorous availability (Plassard et al., 2015). Albeit showing different DH/DC values, three of the four tested strains (PGP27, BS17 and RhOF4) were able to solubilize the three mineral forms of phosphate in TCP medium containing NH_4_Cl or KNO_3_, which might indicate that they use more than one mechanism to solubilize complex phosphate.

Although the plate method is suitable for the first screening of phosphate solubilizing bacteria (Alikhani et al., 2006), phosphate solubilization in liquid media might lead to different results (Hamdali et al., 2008). Indeed, the rhizobial strains RhOF4 and RhOF155, which behaved poorly on NBRIY plates containing tricalcium phosphate, gave better values for soluble phosphorus in liquid medium containing tricalcium phosphate than BS17 and PGP27 strains. This might be due to exopolysaccharides production which could be indirectly involved in phosphate solubilization. Yi et al. (2008) reported that three exopolysaccharides (EPS) producing strains had stronger phosphate solubilization ability than an isolate that did not produce EPS. Moreover, the same study revealed a synergistic effect of exopolysaccharides and organic acids on phosphate solubilization. The lower pH values of the medium observed in our study suggested that phosphate solubilization might be due to the production of organic acids. It is well established that organic acids are involved in the solubilization of phosphate (Ahemad and Kibret, 2014).

Potassium is another macronutrient whose concentration in the soil might be insufficient for optimal plant growth (Parmar and Sindhu, 2013). The strains tested here were able to grow with mica as a potassium source, a characteristic that might be useful for plant nutrition. Han et al. (2006) showed that the application of plants with rock phosphate and rock potassium in combination with inoculation with phosphate and potassium solubilizing bacteria improved mineral availability and concomitantly plant growth.

In addition to their mineral solubilization capacity, the tested strains are also able to produce IAA. Its secretion is a good capability of soil bacteria, including symbionts, which influences bacteria–plant interactions (Souza et al., 2015).

Altogether, the analyzed strains are good candidates for inoculation of plants in the open field. For this reason, we have examined the effect of PGP and rhizobia strains alone or in combination on growth and phosphorus uptake of faba beans and wheat. The inoculation significantly enhanced shoot and root dry weight of bean and wheat plants as well as the weight of bean pods and wheat spikes. Moreover, the phosphorus concentration was higher in inoculated plants. In general, inoculation by rhizobia alone or by a combination of PGP strains and rhizobia gave better results than the treatment with PGP alone. Beside their capacities of phosphate solubilization, we referred these plant improvements to their different PGPR traits useful for plant growth. Several reports studied the advantage of co-inoculation for growing plants. Sánchez et al. (2014) found that native *Rhizobium*-*Pseudomonas* co-inoculation as compared to single *Rhizobium* inoculation increased growth and nodulation of *Phaseolus vulgaris* under Cuban field conditions. Similarly, chickpea grown in laterite soil was greatly enhanced by inoculation with the mixture of a *Rhizobium* and phosphobacterin (*Pseudomonas striata*), especially if phosphate fertilizer was applied (Dutta and Bandyopadhyay, 2009). Bourion et al. (2018) have highlighted that single inoculation experiments can evaluate the ability of a strain to form an effective symbiosis with a given host, but can never determine its ability to compete with other strains in a mixture, which is the circumstance frequently faced in the field. The adaptation of the strains to natural microbiota in the field and their symbiotic associations with the plant should be then taken into consideration for growth improvement in field conditions. On the other hand, some strain combinations might negatively affect the plant while enhancing another or the choice of strains for plant inoculation does not effectively promote the plant growth. For instance, Tsigie et al. (2011) noted that co-inoculation of soybean with *Bradyrhizobium japonicum* and a *Bacillus subtilis* strain increased the growth of soybean. In contrast, these authors have highlighted that this positive effect of co-inoculation could not be observed for lentil. In addition, the greenhouse experiment conducted by Valverde et al. (2006) using *Pseudomonas jessenii* and *Mesorhizobium ciceri* resulted in a decrease in shoot dry weight of chickpea (*Cicer arietinum* L.) with respect to the inoculation with *M. ciceri* alone. While the field study of Verma et al. (2012) indicated that co-inoculation with *Mesorhizobium* sp. BHURC02 and *Bacillus megaterium* do not give comparable results regarding the growth of chickpea as inoculation with *Mesorhizobium* sp. BHURC02 and *Pseudomonas fluorescens*.

Interestingly, the strains used in this study had the ability to adapt to faba bean and wheat rhizospheres while promoting their growth, something that encourages their possible application in large field environments as potential biofertilizers in sustainable agricultural practices. Testing these strain combinations in another environment, with the same and/or other plant species, and in a large-scale field, will provide an interesting perspective for the validation of the obtained results.

## MATERIALS AND METHODS

### Strain isolation

PGP strains were isolated from faba bean and wheat rhizosphere in a field located in the Marrakech-Haouz region (Morocco). 10 g of the soil sample were transferred into flasks containing 90 ml of sterile physiological water and kept on the rotatory shaker at 150 rpm for 30 min. Using the serial dilution technique, bacterial suspensions ranging from dilutions 10^−1^ to 10^−9^ were streaked on The National Botanical Research Institute's phosphate growth medium devoid of yeast extract (NBRIY) (Nautiyal, 1999), containing 5 g/l of tricalcium phosphate: Ca_3_(PO_4_)_2_ as complex form of mineral phosphate. The pH of the culture medium was adjusted to 7 before autoclaving. In order to confirm phosphate solubilizing capacity, the colonies producing a clear halo were purified by repeated streaking on the same medium. As a final step, purified isolates were grown on trypticase soy agar (TSA) at 28°C (Sigma-Aldrich) and were conserved at −20°C in glycerol (25%). The rhizobial strains were isolated according to the method described by Benidire et al. (2018).

### Qualitative estimation of phosphate solubilization in agar media

Four rhizobacterial strains of our collection: RhOF4, RhOF155, BS17 and PGP27, were qualitatively tested on the three agar media: NBRIY (Nautiyal, 1999), TCPNH_4_CL and TCP KNO_3_, containing 5 g/l of three complex phosphate forms: Moroccan rock phosphate, monocalcium phosphate and tricalcium phosphate. TCPNH_4_Cl medium consists of glucose 10 g/l, MgSO_4_^.^7H_2_O 1 g/l, NH_4_Cl 5 g/l, NaCl 1 g/l. TCPKNO_3_ medium contains glucose 10 g/l, MgSO_4_^.^7H_2_O 1 g/l, KNO_3_ 5 g/l and NaCl 1 g/l. Moroccan rock phosphate is a natural mineral form of calcium apatite, Ca_5_(PO_4_)_3_(OH). The chemical composition of Moroccan rock phosphate used in this study was: 56.53% O, 16.35% Ca, 9.37% P, 2.42% F, 2.03% Al, 1.94% Mg, 1.81% Na, 0.77% S, 0.60% Fe, and 0.12% Sn (Hamdali et al., 2008).

Strains were grown first in Erlenmeyer flasks containing 100 ml of trypticase soy broth (TSB) (Sigma-Aldrich) for PGP strains and 100 ml of yeast extract mannitol (YEM) broth for rhizobia strains and incubated at 28°C on a rotary shaker at 180 rpm. Cultures were washed three times to eliminate the residual phosphate attached to bacterial cells and were suspended in a volume of sterile physiological water (distilled water with 9 g/l of NaCl) to reach a final OD_600_ of 0.8.

Using the drop plate method (Alikhani et al., 2006), 7 µl of each strain were spot-inoculated in three biological replicates and incubated at 28°C. The diameters of the halo (DH) and the colony (DC) were measured after 3, 10 and 15 days of incubation. The results were expressed as DH/DC ratio.

### Quantitative estimation of phosphate solubilization in the liquid medium

The strains were grown in 100 ml of TSB (PGP strains) or in 100 ml of YEM broth (rhizobial strains) for 72 h at 28°C and 180 rpm. Strains were washed three times and resuspended in an adequate volume of sterile physiological water in order to obtain an OD_600_ of 0.1. 200 µl of each strain were transferred into 100 ml of NBRIY broth and incubated at 28°C at 180 rpm.

Aliquots of each bacterial culture were taken every 24 h up to 168 h. The supernatant was separated from the bacterial cells by centrifugation at 6000 rpm for 10 min and subsequent filtration (0.2 µm filter). The pH was determined by a glass electrode. Soluble phosphate was measured using the colorimetric method based on the reduction of a phosphorus-molybdate complex (Olsen and Sommers, 1982). The reduction of phosphorus-molybdate is accompanied by a blue coloring whose intensity is proportional to the amount of phosphorus present in the medium. 1 ml of the filtrate was taken and mixed with 4 ml of distilled water and 5 ml of reagent AB [A: sodium molybdate 2.5 g/100 ml H_2_SO_4_ (10 N); B: hydrazine sulfate 0.15 g/100 ml of distilled water], the mixture was incubated for 10 min at 50°C. After cooling samples, the optical density was read on a spectrophotometer at 825 nm. The experiments were done in three biological replicates and the quantity of available phosphate was calculated from a standard curve that was prepared with a solution of KH_2_PO_4_ (Sigma-Aldrich) with variant concentrations ranging from 0 to 2 mg/l in triplicate and handled in the same way as the inoculated samples.

### Indole acetic acid (IAA) production

Strains were grown in 100 ml of Luria Bertani broth (LB) containing 1.02 g/l of L-tryptophan as a precursor of indole acetic acid. After incubation for 4 days at 28°C, strains were washed three times as described above and resuspended in an adequate volume in order to obtain an initial OD_600_=0.1.

Bacterial cells were removed by centrifugation (6000 rpm for 3 min). 1 ml of the supernatant was mixed with 2 ml of Salkowski reagent (10 mM FeCl_3_, 35% perchloric acid) and two drops of orthophosphoric acid were added in three biological replicates. The mixture was incubated in the dark at room temperature for 30 min. The appearance of the red color indicates the presence of IAA produced by the bacteria (Bano and Musarrat, 2003). For quantification, the absorbance at 530 nm was measured using uninoculated LB medium handled in the same conditions as a baseline (zero of absorbance). The amount of IAA was calculated from a standard curve of indole-3-acetamide (Sigma-Aldrich) containing different concentrations from 10 to 60 µg/ml and handled in the same way as described above in triplicate.

### Exopolysaccharide production

Starting from a single colony, a strain was incubated in 10 ml of YEM broth (for rhizobial strains) or 10 ml of TSB (for PGP strains) for 48 h at 28°C. After incubation, cell density was determined by measuring the optical density at 600 nm. 1 ml of each strain was transferred into an Eppendorf tube and Congo Red (CR) was added in order to obtain a final concentration of 40 μg/ml, in three biological replicates. After agitation for 2 h and centrifugation at 14,000 rpm for 5 min, the OD of the supernatant was measured at 490 nm (Spiers et al., 2003). The amount of CR remaining in the supernatant was determined by reference to a standard curve of CR (Lee et al., 2007). Finally, the results were expressed in mg of CR linked to exopolysaccharides divided by bacterial density measured at 600 nm (mg of CR/OD_600_).

### Potassium solubilization

The capacity of potassium solubilization was analyzed as described for phosphate solubilization capacity using the drop method described by Alikhani et al. (2006). Briefly, strains were first grown on TSB or YEM (for rhizobia) at 28°C for 3 days and then washed with sterile physiological water and resuspended in an adequate volume to obtain a final OD_600_ of 0.8.

Alexandrov medium was prepared in 1 l of deionized water containing: 5 g glucose, 0.5 g MgSO_4_^.^7H_2_O, 0.1 g CaCO_3_, 0.006 g FeCl_3_, 2 g Ca_3_PO_4_, 3 g insoluble Mica powder as potassium source and 15 g agar (Parmar and Sindhu, 2013). The agar plates were spot-inoculated with rhizobia or PGP strains in four independent biological replicates. The results were measured after 48 h, 96 h and 144 h and were expressed as the ratio of halo diameter/colony diameter (DH/DC).

### Molecular identification of phosphate solubilizing strains

#### DNA extraction

DNA was isolated as described by Benidire et al. (2018). Strains were grown in TSA medium at 28°C for 2 days. About 4 ml of the bacterial culture were collected by centrifugation. After washing of the bacterial biomass once with TE buffer (10 mM Tris, 1 mM EDTA, pH 8), strains were resuspended in 300 µl TE buffer. 100 µl of 5% SDS and 100 µl pronase E (2.5 mg/ml in TE buffer pre-incubated for 90 min at 37°C) were added. After mixing, the solution was incubated for at least 1 h up to overnight. Then the DNA was thoroughly sheared using a syringe. The DNA was purified by two extractions with 300 µl of Tris-buffered phenol and one extraction with methylene chloride. DNA was precipitated with 0.1 volume of 3 M sodium acetate and 2.5 volumes of ethanol.

#### PCR amplification of 16S rDNA and sequencing

The 16S rDNA was amplified using primers 16Sa (5′-CGCTGGCGGCAGGCTTAACA-3′) and 16Sb (5′-CCAGCCGCAGGTTCCCCT-3′) (van Berkum and Fuhrmann, 2000). The reaction mixture with a total volume of 50 µl, is composed of bacterial DNA (100 ng), DreamTaq buffer, dNTP (100 pmol), DreamTaq polymerase (1.25U) and sterile Milli-Q water. PCR conditions were: an initial cycle of denaturation at 95°C for 5 min; 30 cycles of denaturation at 95°C for 30 s, annealing at 58°C for 30 s, and extension at 72°C for 1.5 min; and a final extension at 72°C for 10 min. The PCR products were checked by horizontal gel electrophoresis (1% w/v agarose) in Tris-Acetate-EDTA (TAE) buffer. The PCR product was purified by the ‘MEGAquick-spin™ Total Fragment DNA Purification Kit’.

Nucleotide sequencing was carried out by GATC Biotech (Konstanz) on both strands using the same primers that were used for PCR. Phylogenetic analysis was conducted with MEGA version 6 (Tamura et al., 2013).

### Field conditions and experimental design

The impact of the mixed inoculation on plant growth was conducted in a field located in the Haouz plain about 15 km from Marrakech, Morocco (the latitude is 31°54′18″ North, the longitude is 8°02′08″ and the elevation above sea level is 511 m). The regional climate of the experimental site is semi-arid with surface soils regularly undergoing drying-rewetting cycles from the irregular distribution of rainfall. The field is an agricultural land equipped with a drip-type irrigation system, no herbicides and no chemical fertilizers were applied in the previous growing seasons. The soil chemical proprieties were as follows: pH (H_2_O) 8.12; carbon 0.5%; organic matter 0.86%; soluble phosphate (ppm) 57. It is a calcareous soil, poor in organic matter and contains a higher percentage of sand and loam (67.04% and 16.34%, respectively). The electrical conductivity is between 0 and 500 µs/cm.

The experiment had a randomized block design with four treatments in eight replicates per treatment dispersed in eight different blocks: (1) control without inoculation (T), (2) PGP alone (P), (3) rhizobial strains alone (R) and finally (4) PGP-rhizobia (PR) as a mixed inoculum. The dimensions of each elementary block were 1.5 m×0.8 m. Each main block was spaced with 0.4 m from the next block. All treatments were carried out for a simple culture system (wheat alone or faba bean alone). The crops were sown from February to May 2017, and the weeds were controlled manually.

360 homogenous bean seeds (Aguadulce variety) and 240 g of homogenous wheat seeds (Karim variety) were disinfected with 1:3 diluted sodium hypochlorite for bean seeds, and 1:5 for wheat seeds. The Karim variety was chosen because it is widely used in North Africa and Morocco due to its stability, productivity and drought resistance (Rezgui et al., 2000). The Aguadulce variety is a Moroccan variety that was extensively cultivated and occupies about 40% of the total area in Morocco (approximately 197,000 ha) (MADRPM/DERD, 2011).

After several series washing with sterile distilled water, seeds were germinated in the laboratory for 48 h up to 72 h for bean seeds and 24 h for wheat seeds. The sprouted seeds were inoculated with (1) PGP alone (PGP27+BS17 at a 1:1V/V ratio), (2) rhizobia alone (RhOF4+RhOF155) at a 1:1V/V ratio) or (3) the mixture PGP-rhizobia (PGP27+BS17+RhOF4+RhOF155), for 30 min in darkness before sowing. The inoculums of PGP or rhizobia were prepared by growing every strain in TSB for the PGP strains at 28°C for one day or YEM broth for the rhizobial strains at 28°C for 2–3 days. The final optical density at 600 nm is equivalent to 1 (approximately 10^9^ CFU/ml).

Based on their size uniformity, 12 homogenous faba bean seeds and 5 g of homogenous wheat seeds (an average of 96 seeds), were transferred into the field. Faba bean seeds were transferred to the field in three rows separated by 0.3 m (four seeds per row), and wheat seeds were sown randomly into each block as it was done by the farmers. A second inoculation by the same combinations was done after 15 days of seed sowing. The second inoculation was carried out with 5 ml of bacterial consortium near the plants' roots (prepared in the same manner as described above).

The harvest was done at the fructification stage. In order to determine the dry weight, shoots and roots of wheat and faba bean were oven-dried at 70°C for 72 h. Green bean pods and dry wheat spikes were also collected, counted and weighed. After drying, shoots and roots were ground and ashed at 550°C. 3 ml of HCl (6N) were added to every sample and directly placed in a hot plate for further evaporation at 330°C for at least 1 h. Finally, 3 ml of hot distilled water was added. The obtained solutions were filtered using Whatman paper of 0.45 µm pore size, the extracts were added to 20 ml of distilled water and stored at 4°C until the determination of phosphorus concentration as described by Olsen and Sommers (1982). 5 ml of samples were added to 5 ml of reagent AB [A: sodium molybdate 2.5 g/100 ml H_2_SO_4_ (10 N); B: hydrazine sulfate 0.15 g/100 ml of distilled water]. The mixture was incubated for 10 min at 50°C. The reduction of phosphorus-molybdate is accompanied by a sky-blue coloration whose intensity is proportional to the amount of phosphorus present in the sample. After 5 min of cooling, the optical density of samples was read at room temperature on a spectrophotometer at λ=825 nm. The amount of phosphorus produced was calculated from a standard curve of KH_2_PO_4_ (Sigma-Aldrich).

### Statistical analysis

We used a completely random block assay design. Growth values of strains are means of three or four biological replicates per treatment for the *in vitro* tests. Concerning the field experiment, results are means of eight replicates per treatment. All the statistical analyses were performed by the analysis of variance (ANOVA) with a least significant difference (LSD) for the comparison of means using COSTAT software. Results are compared via the SNK test (Student, Newmann, Keuls). Means and standard deviations are presented in the graphs. Means with different letters are significantly different at *P*<0.05.
